# Improving the Quality of Surgical Operative Notes at Managil Teaching Hospital, Sudan: A Clinical Audit

**DOI:** 10.7759/cureus.92214

**Published:** 2025-09-13

**Authors:** Abubakr Muhammed, Abdalmahmoud Asadig Kanan Ahmed, Amir Malik Ibrahim Algak, Sara Omer Mohamed Abdalla, Mohamed Elhassan Momin Mohamed Elhassan Noreldayem, Shima Mohamed, Tesnim Abdon, Mohamed Abdalrhman Nour Ahmed, Khadega Osman Abbas Alhudi, Hisham Osman Adam Suliman, Romisaa Elamin, Mohamed Shamseldin Elsiddig Mohamed, Maysara Elsiddig, Asjed Ahmed, Asim Faisal Awad Ibrahim, Khalid Badreldin Awadelkarim Mahmoud, Ali Shamsaldeen, Sara Y Mukhtar, Shima Abdalraheem Elnadeef Hussein, Moiez Mohammed Aboudi Ahmed

**Affiliations:** 1 Surgery, University of Gezira, Madani, SDN; 2 General Surgery, Managil Teaching Hospital, Managil, SDN; 3 General Surgery, Burjeel Medical City, Abu Dhabi, ARE; 4 Paediatric Surgery, Royal Manchester Children's Hospital, Manchester, GBR; 5 Trauma and Orthopaedics, Rotherham General Hospital, Rotherham, GBR

**Keywords:** clinical audit, documentation, quality improvement projects, surgery general, surgical operative note

## Abstract

Background: Accurate and comprehensive surgical operative notes are crucial for patient safety, continuity of care, and medico-legal purposes. However, inconsistencies in documentation quality at Managil Teaching Hospital, Sudan, prompted a clinical audit to evaluate adherence to international standards.

Materials and Methods: This study employed a retrospective-prospective clinical audit design conducted in the General Surgery Department at Managil Teaching Hospital. We evaluated a total of 101 operative notes (51 in the first, retrospective cycle from May 15 to June 15, 2025, and 50 in the second, prospective cycle from June 16, 2025, to July 16, 2025). Notes were assessed against 18 parameters derived from the Royal College of Surgeons of England (RCS) guidelines using a standardized checklist. Interventions included the introduction of a standardized operative note template, staff training sessions on best practices, and the display of visual aids in operating theaters. Data were systematically analyzed using Microsoft Excel 2016 (Microsoft Corp., Redmond, WA), employing descriptive statistics to compare compliance rates and calculate percentage improvements between cycles.

Results: The audit revealed significant improvements in documentation quality following interventions. Notably, there were substantial gains in recording tissue details (from 25 (49%) to 42 (84%), an 35% improvement), prostheses (from 20 (39.2%) to 38 (76%), a 36.8% improvement), and postoperative instructions (from 40 (78.4%) to 45 (90%), an 11.6% improvement). The most significant improvements were observed in documenting complications (15 (29.4%) to 35 (70%), +40.6%) and extra procedures (10 (19.6%) to 30 (60%), +40.4%). However, some gaps persisted, particularly in recording anticipated blood loss (10 (19.6%) to 25 (50%)) and DVT prophylaxis (15 (29.4%) to 30 (60%)).

Conclusion: At Managil Teaching Hospital, the adoption of structured approaches like standardized templates, specialized training, and visual aids significantly boosted the caliber of surgical operative notes. Despite this considerable improvement, persistent vigilance, ongoing education, and consistent audits are crucial to tackle lingering weaknesses, particularly in documenting DVT prophylaxis and projected blood loss, thereby ensuring sustained enhancement in quality.

## Introduction

Thorough and precise surgical operative notes are absolutely essential. They form the bedrock of patient safety, facilitate effective postoperative care, and are crucial for maintaining legal accountability [1]. They serve as a critical communication tool among healthcare providers and ensure continuity of care. However, in many low-resource settings, including Sudan, operative documentation often lacks standardization, leading to incomplete or inconsistent records [2]. Poor documentation can compromise patient outcomes, hinder clinical audits, and increase medico-legal risks [3]. 

The Royal College of Surgeons of England (RCS) has established guidelines for operative notes, emphasizing essential elements such as patient identification, procedural details, complications, and postoperative instructions [4]. Adherence to these standards ensures clarity, completeness, and legal defensibility. Despite their importance, studies in Sudanese hospitals indicate persistent deficiencies in operative documentation, particularly in recording complications, prosthesis details, and prophylactic measures [5]. 

The objective of this clinical audit at Managil Teaching Hospital was to assess the baseline quality of surgical operative notes against the RCS guidelines, implement structured interventions to improve documentation standards, and subsequently re-evaluate outcomes to determine the extent of improvement and ensure sustained compliance with best practice.

## Materials and methods

Study design and setting

A retrospective-prospective clinical audit was conducted in the General Surgery Department at Managil Teaching Hospital, Sudan. The study spanned two cycles. The first cycle (retrospective) evaluated 51 operative notes from May 15 to June 15, 2025. The second cycle (prospective) assessed 50 notes following interventions from June 16 to July 16, 2025.

Audit population and sample size

This clinical audit was conducted within the General Surgery Department at Managil Teaching Hospital, Sudan. The audit population comprised surgical operative notes generated during two distinct periods. For the first cycle (retrospective), a sample of 51 surgical operative notes was evaluated, collected using total coverage sampling of procedures performed from May 15 to June 15, 2025. Subsequently, for the second cycle (prospective), a sample of 50 operative notes was assessed, representing documentation created after the implementation of interventions, from June 16 to July 16, 2025. In both cycles, efforts were made to ensure the samples were identical or almost identical in characteristics to minimize any potential sampling bias. To further strengthen transparency and reproducibility, consecutive operative notes were included without exclusion criteria to avoid selection bias and ensure comparability across both audit cycles.

Data collection

Operative notes were meticulously reviewed using a standardized checklist specifically derived from the RCS guidelines [1]. This comprehensive checklist assessed 18 key parameters to ensure a thorough evaluation of documentation quality. These parameters included essential details such as the date and time of the operation, type of operation (elective/emergency), names of the surgeon, assistant, and anesthetist, operative procedure, incision type, operative diagnosis, and operative findings. Furthermore, the checklist covered documentation of complications, extra procedures and their rationale, details of tissue removed or altered, prostheses or materials used, closure technique, anticipated blood loss, antibiotic prophylaxis, deep vein thrombosis (DVT) prophylaxis, postoperative instructions, and the surgeon's signature [1]. The use of this validated checklist ensured standardization and minimized observer bias during data collection.

Data analysis

The data gathered for this study underwent a thorough examination using Microsoft Excel 2016 (Microsoft Corp., Redmond, WA). Our primary method of analysis involved descriptive statistics, where we pinpointed the compliance rates for all 18 parameters during both the initial and subsequent audit periods. To truly grasp the influence of our interventions, we then computed the percentage improvement for each individual parameter. This was achieved by directly contrasting the compliance rates from the first audit cycle with those from the second. This methodical approach provided a clear and quantifiable measure of how effective our implemented strategies were in elevating the standard of surgical operative notes [1]. The choice of descriptive statistics was appropriate, given the audit design and the categorical nature of compliance data, while Excel was deemed adequate for ensuring accurate and transparent calculations.

Interventions

Following the initial cycle, several key interventions were implemented. A standardized operative note template was introduced, designed in alignment with the RCS guidelines [1]. This layout emphasized clarity and ease of use, with dedicated spaces for each parameter to minimize omissions.

Training sessions were also conducted for consultants, registrars, and junior surgical staff. These sessions included interactive workshops on the importance of accurate operative documentation, the medicolegal implications of incomplete notes, and the role of high-quality records in continuity of care and audit cycles.

## Results

The clinical audit conducted across two cycles revealed substantial improvements in the documentation of surgical operative notes. Several key parameters showed enhanced compliance with established standards following targeted educational interventions.

There was a notable improvement in the documentation of date and time (45 (88.2%) to 48 (96%)), ensuring precise record-keeping for operative procedures. The type of operation recorded rose from 30 (58.8%) in the first cycle to 42 (84%) in the second cycle, reflecting better identification of procedural details. The documentation of the surgeon and assistant names improved from 47 (92.2%) to 49 (98%), reinforcing accountability within surgical teams.

An increased number of operative notes included the anesthetist's name, growing from 28 (54.9%) to 35 (70%), demonstrating better recognition of multidisciplinary involvement. The operative procedure details showed a consistent enhancement, reaching 48 (96%) in the second cycle, while incision type documentation improved significantly from 32 (62.7%) to 42 (84%).

The operative diagnosis saw a marked increase in compliance from 40 (78.4%) to 45 (90%), ensuring more precise identification of surgical indications. Additionally, operative findings documentation improved from 35 (68.6%) to 42 (84%), aiding in more comprehensive surgical records.

One of the most significant improvements was observed in the documentation of complications, rising from 15 (29.4%) to 35 (70%), demonstrating enhanced awareness of postoperative risks. Extra procedures recorded increased from 10 (19.6%) to 30 (60%), ensuring additional interventions were properly documented. Similarly, the inclusion of tissue details improved dramatically from 25 (49%) to 42 (84%), while prostheses documentation saw an increase from 20 (39.2%) to 38 (76%), ensuring thorough reporting of implanted devices.

Further improvements were observed in closure technique (30 (58.8%) to 42 (84%)) and anticipated blood loss (10 (19.6%) to 25 (50%)), emphasizing better intraoperative assessments. The documentation of antibiotic prophylaxis grew from 35 (68.6%) to 42 (84%), supporting adherence to infection prevention protocols, while DVT prophylaxis increased from 15 (29.4%) to 30 (60%), ensuring preventive measures were properly recorded.

Postoperative instructions showed improvement from 40 (78.4%) to 45 (90%), reinforcing the importance of clear postoperative care directives. Finally, the surgeon’s signature compliance improved from 45 (88.2%) to 48 (96%), reflecting better adherence to procedural accountability.

Overall, the audit demonstrated significant progress across all assessed parameters, highlighting the effectiveness of structured interventions in improving surgical documentation standards. Continued re-auditing and educational initiatives are recommended to sustain these improvements and further optimize operative record-keeping practices.

 The audit demonstrated significant improvements in most documentation parameters (Table 1). 

**Table 1:** Compliance Rates Before and After Intervention DVT: deep vein thrombosis

No.	Parameter	First Cycle (N=51)	Second Cycle (N=50)	Improvement (%)
1.	Date and time	45 (88.2%)	48 (96%)	7.8%
2.	Type of operation	30 (58.8%)	42 (84%)	25.2%
3.	Surgeon and assistant name	47 (92.2%)	49 (98%)	5.8%
4.	Anesthetist’s name	28 (54.9%)	35 (70%)	15.1%
5.	Operative procedure	45 (88.2%)	48 (96%)	7.8%
6.	Incision type	32 (62.7%)	42 (84%)	21.3%
7.	Operative diagnosis	40 (78.4%)	45 (90%)	11.6%
8.	Operative findings	35 (68.6%)	42 (84%)	15.4%
9.	Complications	15 (29.4%)	35 (70%)	40.6%
10.	Extra procedures	10 (19.6%)	30 (60%)	40.4%
11.	Tissue details	25 (49%)	42 (84%)	35%
12.	Prostheses documentation	20 (39.2%)	38 (76%)	36.8%
13.	Closure technique	30 (58.8%)	42 (84%)	25.2%
14.	Anticipated blood loss	10 (19.6%)	25 (50%)	30.4%
15.	Antibiotic prophylaxis	35 (68.6%)	42 (84%)	15.4%
16.	DVT prophylaxis	15 (29.4%)	30 (60%)	30.6%
17.	Postoperative instructions	40 (78.4%)	45 (90%)	11.6%
18.	Surgeon’s signature	45 (88.2%)	48 (96%)	7.8%

## Discussion

Progress in documentation quality 

This audit demonstrated substantial improvements in surgical note completeness following structured interventions. The introduction of a standardized template and training sessions significantly enhanced documentation of critical elements such as complications, extra procedures, and prostheses. These findings align with similar audits in Sudanese hospitals, where standardized proformas improved compliance rates by 20-40% [6]. 

Comparison with Sudanese studies 

Studies at Dongola Teaching Hospital and Port Sudan Teaching Hospital reported comparable challenges, particularly in documenting DVT prophylaxis and blood loss [7, 8]. Similarly, a two-phase audit at Elobeid Teaching Hospital revealed baseline deficiencies in recording anticipated blood loss (0%), DVT prophylaxis (2%), and postoperative instructions (34%). However, after implementing a standardized template and staff training, compliance rates surged to 97-100% for key parameters like antibiotic prophylaxis and operative findings [9]. Another audit at Port Sudan Teaching Hospital demonstrated a rise in overall documentation compliance from 51.9% to 82.1%, with notable improvements in DVT prophylaxis (4% to 98%) and complications documentation (30% to 98%) [10]. These findings align with our results, underscoring the systemic nature of documentation gaps in Sudanese hospitals and the effectiveness of structured interventions. 

Our intervention-driven improvements (e.g., +40.6% in complications documentation) exceed those seen in prior audits, highlighting the added value of targeted training and visual aids. However, persistent gaps in DVT prophylaxis (60%) and anticipated blood loss (50%) mirror trends observed across these studies, suggesting that these areas require focused reinforcement. 

International context 

Globally, audits in Malawi and Pakistan have shown that electronic templates and regular feedback can achieve >90% compliance [11, 12]. While our study did not reach this level, the improvements are notable given resource constraints. The persistent gap in DVT prophylaxis documentation (60%) reflects broader challenges in low-resource settings, where postoperative protocols are often inconsistently applied [13]. 

Limitations 

The study's findings, derived from a single-center study, might not be broadly applicable to other healthcare facilities due to variations in practices and patient populations. Additionally, the short follow-up period means that the long-term sustainability of any observed improvements remains unconfirmed. Finally, while informative, the sample size of this audit suggests that larger-scale investigations could potentially uncover further areas for improvement. 

## Conclusions

This audit confirmed that structured interventions - standardized templates, training, and visual aids - significantly improve surgical operative note quality at Managil Teaching Hospital. Though progress was made, ongoing efforts are needed to address persistent gaps, particularly in DVT and blood loss documentation. Regular audits and digital solutions should be explored to sustain improvements. 

## References

[1] (2025). Royal College of Surgeons of England. Good Surgical Practice. Good Surgical Practice.

[2] World Health Organization (2009). WHO Guidelines for Safe Surgery 2009.

[3] Parwaiz H, Perera R, Creamer J, Macdonald H, Hunter I (2017). Improving documentation in surgical operation notes. Br J Hosp Med (Lond).

[4] Mohamed A, Abdalla M (2024). A study in Wad Madani, Sudan: are we documenting operation notes effectively?. Cureus.

[5] Elhadi Bakheet O, Muhammed A, Mohamed A (2024). Evaluation and improvement of the quality of surgical operative notes in the Department of General Surgery at Dongola Teaching Hospital, Sudan. Cureus.

[6] Khalid A, Shahzad MZ, Ahmed H, Gilani A, Khan KH (2022). Audit of operative notes against Royal College of Surgeons guidelines in a tertiary health care surgical unit in Lahore. Cureus.

[7] Hassan Ibrahim HM, Abdelrahman Zeineldeen AA, Mohamed Mohamed Alameen MA (2024). Enhancing surgical operative note standards at Doka hospital, Sudan: a clinical audit. Cureus.

[8] Toru HK, Aizaz M, Orakzai AA, Jan ZU, Khattak AA, Ahmad D (2023). Improving the quality of general surgical operation notes according to the Royal College of Surgeons (RCS) guidelines: a closed-loop audit. Cureus.

[9] Muhammed A, Ibrahim Mohamed AM, Awad Ibrahim AF (2025). Evaluating and improving the quality of surgical operative notes at Elobeid Teaching Hospital: a two-phase audit. Cureus.

[10] Abdelbagi AY, Muhammed A, Elamin Elnour MA (2024). Evaluating and improving the quality of surgical operative notes at the Port Sudan Teaching Hospital. Cureus.

[11] Nyamulani N, Mulwafu W (2018). The quality of hand-written operative notes in a surgical unit at Queen Elizabeth Central Hospital (QECH), Malawi: A prospective completed audit loop study. Malawi Med J.

[12] Qasem M, Qasem O, Skalidi N (2024). Improving the quality and standardization of operative notes in a tertiary regional ENT department: a closed-loop audit. Cureus.

[13] Babalola RN, Olasehinde O, Sowande OA (2016). An audit of the quality of surgical operation notes in a Nigerian teaching hospital. East Cent Afr J Surg.

